# Combined Extra- and Intrathoracic (Dumbbell) Lipoma Crossing the Third Intercostal Space

**DOI:** 10.7759/cureus.96353

**Published:** 2025-11-08

**Authors:** Magdalena Alexieva, Georgi Gergov, Evgeni V Mekov, Elitsa Yankova, Georgi Yankov

**Affiliations:** 1 Thoracic Surgery Department, Medical University - Sofia, Sofia, BGR; 2 Pulmonary Disease Department, Medical University - Sofia, Sofia, BGR

**Keywords:** chest wall, dumbbell lipoma, intrathoracic lipoma, resection, treatment

## Abstract

Intrathoracic lipomas are uncommon, and dumbbell (hourglass) lesions with contiguous extra- and intrathoracic components are exceedingly rare. Surgery is indicated for enlarging or symptomatic tumors or when imaging raises concern for malignancy (e.g., infiltration). Complete resection - via thoracotomy or video-assisted thoracoscopic surgery (VATS) - provides definitive histopathologic diagnosis, cures benign disease, and helps prevent future compressive complications. We report a 53-year-old woman with a combined extra- and intrathoracic lipoma managed with a left oblique parasternal (subpectoral) incision and uniportal VATS. Complete removal was achieved, and histopathology confirmed lipoma. The postoperative course was uneventful, with discharge on postoperative day four. A brief review of the literature is also provided.

## Introduction

Lipomas are common benign mesenchymal tumors, and the majority of them originate from subcutaneous adipose tissue. Although lipomas can form in almost every organ in the body, more than half of them are found in the subcutaneous regions [1]. They are more often found in people who are obese and between the ages of 40 and 60 [2]. Dumbbell (hourglass) combined extra- and intrathoracic tumors are extremely rare, and for larger lesions, a combined approach - coordinated extra- and intrathoracic access through separate incisions - is often required to mobilize and remove both components safely. Because lipoma and atypical lipomatous tumor/well-differentiated liposarcoma (ALT/WDLPS) can overlap on imaging and even on limited biopsy samples, percutaneous biopsy alone may be falsely reassuring (sampling error can capture only mature adipocytes and miss atypia). When a fat-containing mass is deep, large, or enlarging, many authors recommend complete excision to secure a definitive diagnosis (with MDM2/CDK4 testing when indicated) and treat symptoms [1].

Although lipomas are frequent overall, deep intrathoracic localizations are rare, and pleural lipomas are an exceptionally uncommon subset. Solid pleural tumors have been estimated at ~2.8 per 100,000 hospitalizations, and pleural lipomas are encountered only sporadically on chest CT (six cases among 4,000 scans in one classic series) [3,4]. Moreover, hourglass (dumbbell) lipomas that traverse an intercostal space from the chest wall into the pleural cavity are particularly rare and are described mainly in isolated case reports [5].

## Case presentation

**Figure 1:**
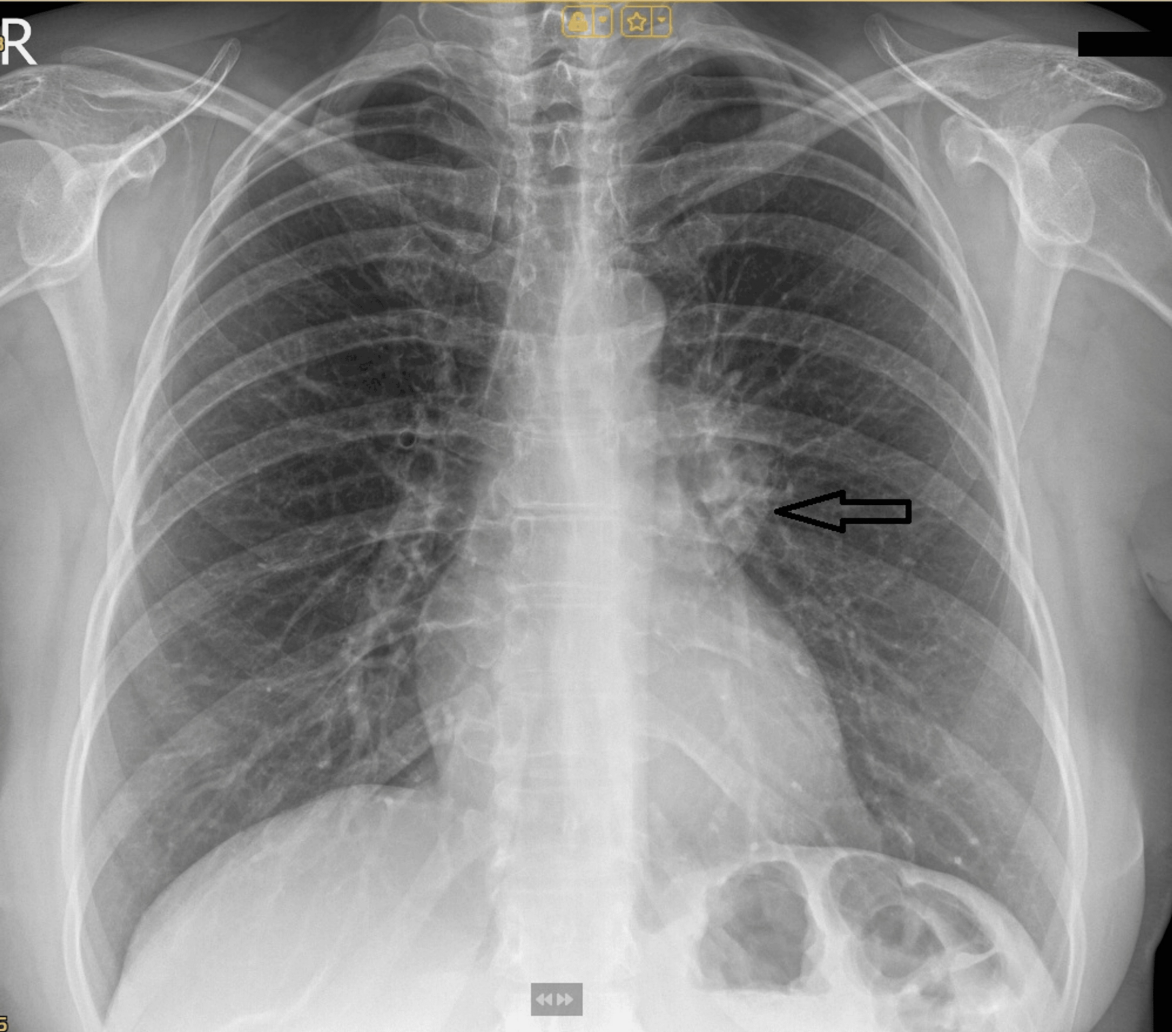
A preoperative chest radiograph (posteroanterior view) demonstrated a subtle, ill-defined left perihilar opacity (black arrow).

**Figure 2:**
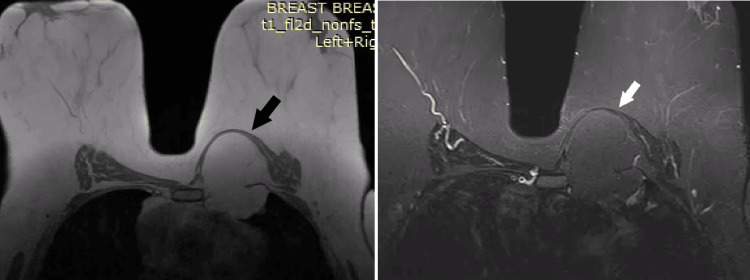
Magnetic resonance imaging of a dumbbell-shaped combined extra- and intrathoracic lipoma. (A) Axial T1-weighted 2D gradient-echo (FLASH), non-fat-suppressed image. (B) Axial T1-weighted fat-suppressed image. White and black arrows indicate the lesion.

A 53-year-old woman was admitted to the Department of Thoracic Surgery with a three-month history of a progressively enlarging mass at the junction of the sternum and the medial left breast. A preoperative chest radiograph (Figure 1) demonstrated a subtle, ill-defined left perihilar opacity. Magnetic resonance imaging (MRI) demonstrated a well-circumscribed, fat-containing lesion consistent with a lipoma located deep to the left pectoralis major muscle (Figure 2). The mass measured 72 × 60 × 65 mm and exhibited a dumbbell (hourglass) configuration. Its extrathoracic component (52 × 48 × 25 mm) traversed between the sternal attachments of the second and third ribs, extending into the left pleural cavity near the anterior mediastinum, where it abutted the right ventricle without evidence of invasion.

The patient reported no known comorbidities and was a current smoker with a 35-pack-year history. On examination, a soft, mildly tender swelling was visible and palpable over the sternal-medial left breast region. Routine laboratory studies were within reference ranges.

Preoperative differential diagnosis based on MRI favored a benign lipomatous lesion (lipoma or fibrolipoma) but included atypical lipomatous tumor/well-differentiated liposarcoma (ALT/WDLPS) given the deep location. Thymolipoma was considered less likely because of the subpectoral origin and trans-intercostal isthmus rather than a thymic pedicle, and there were no features of hernia or non-fatty nodular components on imaging.

**Figure 3:**
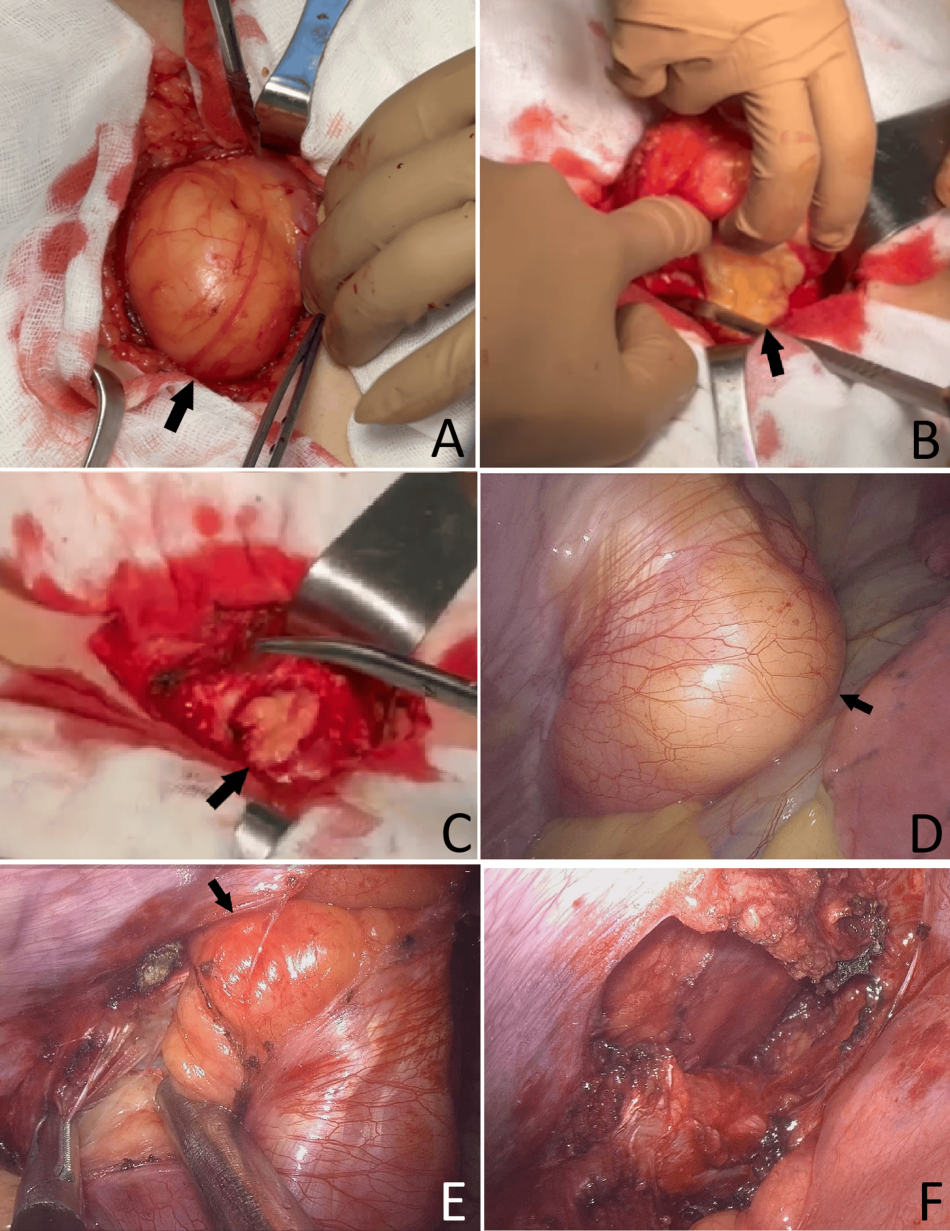
Intraoperative photographs of resection of a dumbbell (hourglass) lipoma. (A) Mobilization of the extrathoracic component. (B) Excision of the extrathoracic component. (C) Residual intrathoracic component viewed externally. (D) Intrathoracic component within the pleural cavity. (E) Thoracoscopic dissection of the intrathoracic lipoma. (F) Final thoracoscopic view after complete resection. Black arrows indicate the lipoma.

A slightly oblique incision was made in the upper inner quadrant of the left breast (Figure 3). The superficial pectoral fascia and the underlying pectoralis major muscle were divided, exposing a well-encapsulated subpectoral (submuscular) lesion with a macroscopic appearance consistent with lipoma. Lateral mobilization revealed a narrow pedicle extending into the left pleural cavity through the third intercostal space, adjacent to the left sternal border.

​​​​We proceeded with a left uniportal video-assisted thoracoscopic surgery (VATS). In the pleural cavity, a well-encapsulated mass was seen descending from the third intercostal space. Using an ultrasonic energy device (harmonic scalpel) and sharp dissection, the intrathoracic component was mobilized circumferentially. The extra- and intrathoracic components were continuous, representing a single dumbbell (hourglass) lesion. Because en bloc removal through either approach was not feasible, the mass was divided at its narrowest isthmus at the level of the third intercostal space. The pleural component was retrieved through the thoracoport, and the extrathoracic component through the chest-wall incision. Both specimens were submitted for intraoperative frozen-section analysis, which showed no evidence of malignancy. Operative time was 40 minutes with negligible estimated blood loss (~0 mL), and there were no intraoperative complications.

**Figure 4:**
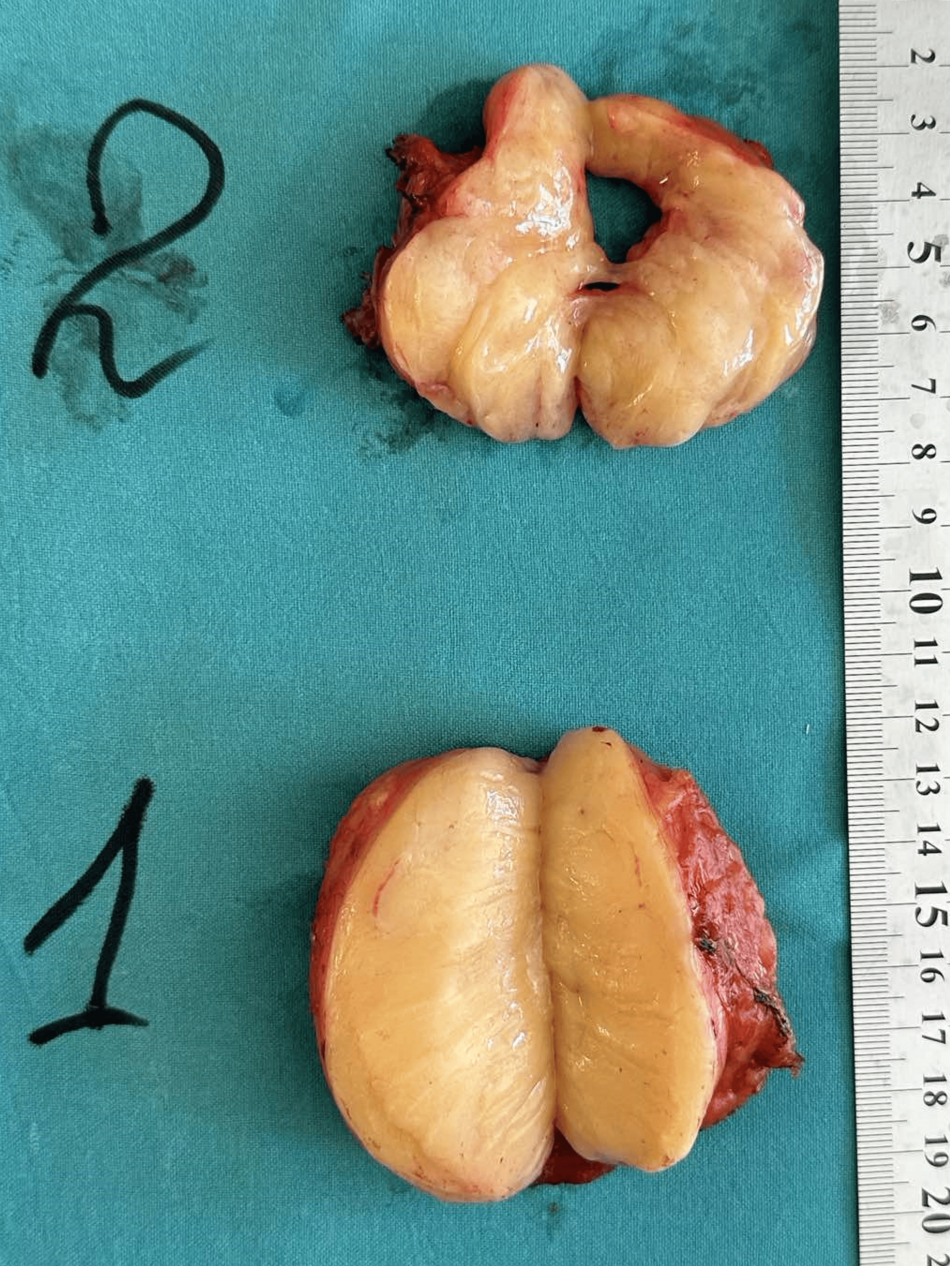
Gross (macroscopic) view of the dumbbell (hourglass) lipoma after transection. 1, extrathoracic component; 2, intrathoracic (pleural) component

 Grossly, the specimen consisted of a well-encapsulated lipoma (Figure 4). The extrathoracic component measured 80 × 60 mm, and the intrathoracic component measured 70 × 35 mm.

**Figure 5:**
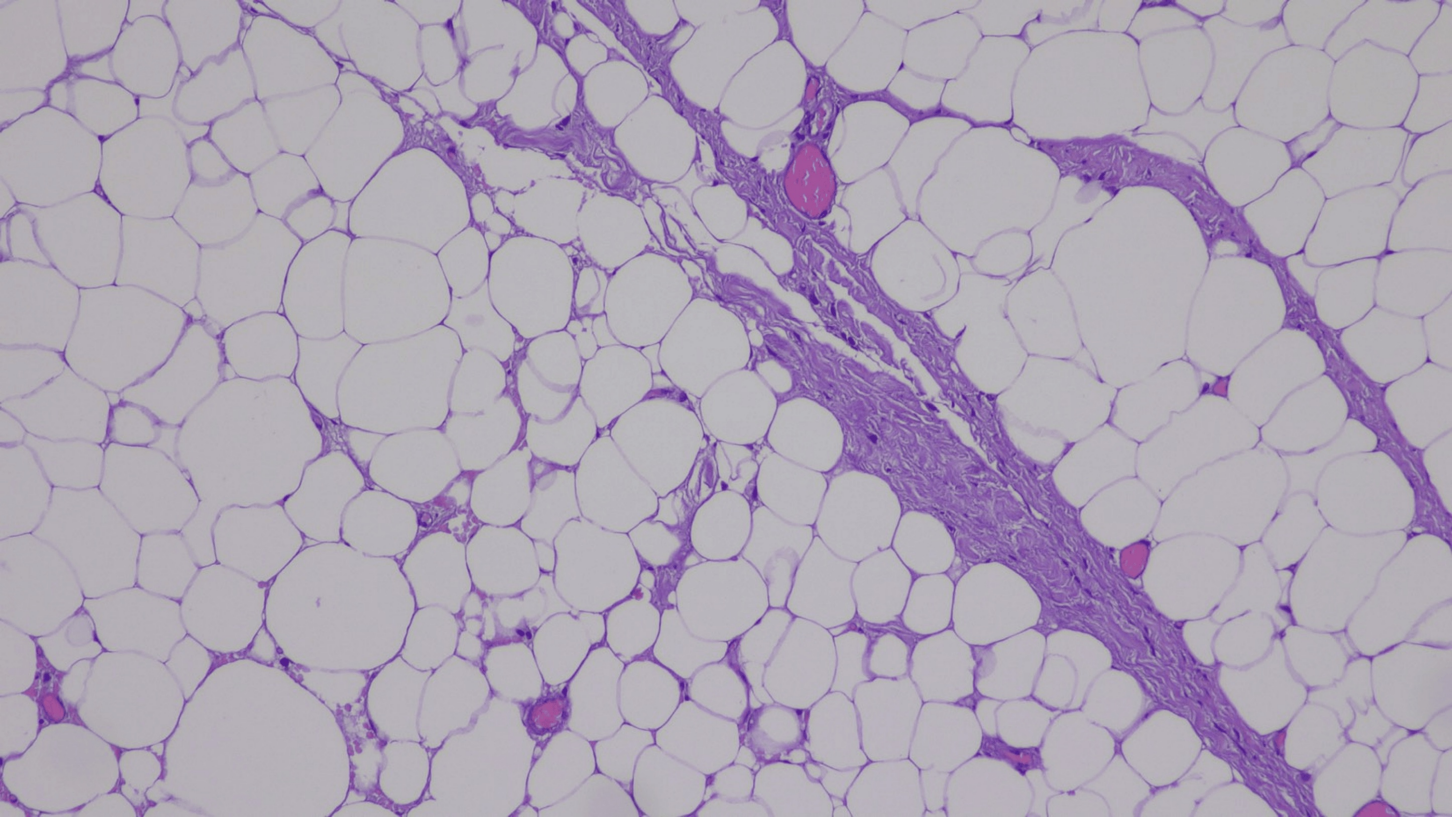
Photomicrograph of the dumbbell (hourglass) lipoma (hematoxylin-eosin stain).

Permanent histopathology confirmed a benign mesenchymal lesion consistent with lipoma (Figure 5).

The postoperative course was uneventful, and the patient was discharged on postoperative day four. At four-month follow-up, there was no evidence of recurrence.

## Discussion

We present a rare dumbbell-shaped lipoma that traverses an intercostal space, managed in one session with a hybrid approach: a subpectoral incision for the extrathoracic component plus uniportal VATS for the intrathoracic component.

Lipomas are the most common benign mesenchymal tumors in adults and are classified by location as superficial or deep [6]. Lipomas account for at least 30% of benign soft-tissue tumors, though true population prevalence is uncertain because many lesions are asymptomatic and never sampled [7]. Deep lipomas are less frequent, tend to be larger, and often arise in the hands, feet, and chest wall; intrathoracic localization is uncommon, with lesions reported at mediastinal, endobronchial, or pulmonary sites [1,2,8].

Intrathoracic lipomas are rare, making up 1.6-2.3% of all mediastinal tumors and 0.1% of all lung tumors [9]. Intrathoracic lipomas present either as entirely intrathoracic masses or as dumbbell (hourglass) lesions extending from the subcutaneous space into the thoracic cavity through an intercostal space or the thoracic inlet [10]. This distinction is clinically consequential because symptom patterns and management differ: purely intrathoracic tumors mainly cause intrathoracic mass effect (e.g., dyspnea), whereas dumbbell lesions often present with a palpable chest-wall component and typically require a combined extra- and intrathoracic approach to achieve complete excision.

Most lipomas enlarge slowly and remain asymptomatic. When symptoms occur, they reflect mass effect and depend on size and location (e.g., dyspnea or dysphagia with airway or esophageal compression) [8,11]. The most common symptoms are exertional dyspnea (~38.9%) and cough (~19.4-30.6%) [12]. In our case, the patient noted a painless swelling over the sternal-medial left breast.

Upon physical inspection, most intramuscular lipomas appear to be circumscribed masses of homogeneous, yellowish adipose tissue with mottled tan areas and soft on palpation [6]. The histological appearance of intramuscular lipoma is characterized by mature, univacuolated adipocytes that are fairly consistent in size and shape, which infiltrate between muscle fibers randomly and, in many cases, totally replace the muscle bundles [6]. Importantly, the absence of cytologic atypia, lipoblasts, and mitotic activity supports a diagnosis of lipoma and helps exclude atypical lipomatous tumor/well-differentiated liposarcoma, but when morphology is equivocal, ancillary MDM2/CDK4 testing is recommended [6]. Deep-seated lipomas, particularly intrathoracic ones, must be carefully distinguished from malignancies such as liposarcomas or metastatic lesions because lipomatous cancers often do not grow in the subcutaneous area [1]. Red flags suggesting malignancy include thick (>2 mm) or nodular septa, non-adipose enhancing nodules, <~75% macroscopic fat, ill-defined margins or invasion of adjacent structures, and rapid interval growth. The most reliable method for diagnosing pleural lipoma is a biopsy [11].

Imaging is central to diagnosis, but cannot always exclude malignancy. Chest radiographs are nonspecific [2]. Characteristic CT findings include a well-circumscribed, homogeneous fat-attenuation mass (−50 to −150 HU), thin septa/capsule, obtuse angles with the chest wall, and displacement rather than invasion of adjacent structures [1]. MRI shows fat-signal intensity on T1/T2 with suppression on fat-sat sequences, supporting a benign lipomatous lesion, although overlap with well-differentiated liposarcoma can persist [6,13]. In our case, MRI demonstrated a well-encapsulated, homogeneous fat-containing mass deep to the left pectoralis major with a narrow intercostal isthmus traversing the third intercostal space into the anterior pleural cavity. Biopsy may be required for pleural lipomas when imaging is equivocal [11]. In the setting of progressive growth and typical fat characteristics, we proceeded directly to resection.

Management is dictated by size, depth, and symptomatology. Observation is reasonable for small superficial lipomas (≤3 cm), subcutaneous lesions that are asymptomatic, non-growing, and demonstrate typical benign fat characteristics on imaging. In contrast, deep or intrathoracic fatty masses are generally managed by complete excision because diagnostic uncertainty persists and compressive complications could develop [6,8,13,14]. VATS is effective for pedunculated, non-infiltrative tumors [8,14]. In our patient, en bloc extraction was precluded by size and configuration; therefore, we divided the lesion at its narrow isthmus and removed the components separately, similar to prior reports [15,16]. Outcomes are favorable after complete excision, with local recurrence rates <5%. Deep location and incomplete resection increase risk [8,17].

This case highlights several practical points for anterior parasternal hourglass lipomas. First, an MRI demonstrating a narrow intercostal isthmus helps anticipate the need for a combined extra- and intrathoracic approach. Second, when the pedicle is too narrow or the angles too acute for en bloc retrieval, controlled division at the isthmus permits complete and safe removal of both components with low morbidity via uniportal VATS. Similar combined strategies for chest wall/pleural hourglass lipomas have been reported, but remain limited to case reports [8,18].

## Conclusions

Dumbbell (hourglass) extra- and intrathoracic lipomas are exceedingly rare. Surgical management is warranted for enlarging or symptomatic lesions, or when imaging raises concern for malignancy (e.g., infiltration or indistinct margins). Complete resection via thoracotomy or VATS provides definitive histologic diagnosis, achieves cure in benign disease, and helps prevent compressive complications and recurrence.
